# A new energy transfer channel from carotenoids to chlorophylls in purple bacteria

**DOI:** 10.1038/s41467-017-00120-7

**Published:** 2017-07-10

**Authors:** Jin Feng, Chi-Wei Tseng, Tingwei Chen, Xia Leng, Huabing Yin, Yuan-Chung Cheng, Michael Rohlfing, Yuchen Ma

**Affiliations:** 1 School of Chemistry and Chemical Engineering, Shandong University, Jinan, 250100 China; 2 Department of Chemistry and Center for Quantum Science and Engineering, National Taiwan University, Taipei, 106 Taiwan; 3 School of Physics and Electronics, Henan University, Kaifeng, 475004 China; 4 Institut für Festkörpertheorie, Universität Münster, Münster, 48149 Germany

## Abstract

It is unclear whether there is an intermediate dark state between the S_2_ and S_1_ states of carotenoids. Previous two-dimensional electronic spectroscopy measurements support its existence and its involvement in the energy transfer from carotenoids to chlorophylls, but there is still considerable debate on the origin of this dark state and how it regulates the energy transfer process. Here we use ab initio calculations on excited-state dynamics and simulated two-dimensional electronic spectrum of carotenoids from purple bacteria to provide evidence supporting that the dark state may be assigned to a new A_g_^+^ state. Our calculations also indicate that groups on the conjugation backbone of carotenoids may substantially affect the excited-state levels and the energy transfer process. These results contribute to a better understanding of carotenoid excited states.

## Abstract (web summary)

Carotenoids harvest energy from light and transfer it to chlorophylls during photosynthesis. Here, Feng et al. perform ab initio calculations on excited-state dynamics and simulated 2D electronic spectrum of carotenoids, supporting the existence of a new excited state in carotenoids.

## Introduction

**Fig. 1:**
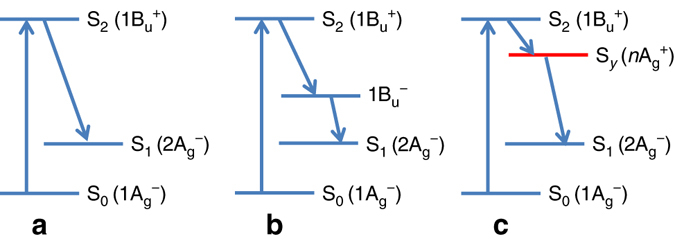
Photophysics model of carotenoids. The three-state model (**a**), the four-state model involving 1B_u_^−^ (**b**) and the new four-state model containing S_*y*_ (**c**) used to interpret the excited-state dynamics of carotenoids

Carotenoids (Cars) take part in various processes in living organisms. In living animals and humans, they act as antioxidants preventing free radicals from destroying tissue cells^1^ and are relevant to the vision of retina^2^. In the photosynthesis of plants and microorganisms, Cars are responsible for harvesting light, transferring energy to chlorophylls (Chls), and protecting against excessive light by quenching excited states of Chls^3, 4^. Exploring the excited-state dynamic properties of Cars is fundamental to understand the mechanism of photosynthesis and is also helpful for developing artificial light-harvesting systems. Despite the studies for several decades, our knowledge on the electronic structure and excited-state properties of Cars, which determine the mechanism of energy decay in Cars and energy flow from Cars to Chls, is still limited. For example, the origin and nature of a dark state, which lies between the strongly one-photon allowed S_2_ (1B_u_^+^) state and the forbidden S_1_ (2A_g_^−^) state, has triggered intense research and has been under debate. This dark state would have a critical role in mediating the Car-to-Chl energy transfer process and the depopulation of the S_2_ state^4–11^. Discovery of this dark state would make the conventional carotenoid photophysics model S_0_→S_2_→S_1_→S_0_ (Fig. 1a), where S_0_ is the ground state 1A_g_^−^, no longer accurate. Therefore, a new model may be required to account for the excited-state behavior of Cars^4, 12, 13^.

A number of experimental and theoretical studies^4, 8, 14, 15^ have been carried out in the past 20 years to unravel this dark state. The 1B_u_^−^ state is the popular choice for the attribution of the dark state (Fig. 1b), and it also seems to be the only choice based on the present theory on the excited-state structure of polyenes, which are closely related to the Cars. However, this assignment is controversial due to the contradiction between the properties of the dark state measured experimentally and the behavior of the 1B_u_^−^ state determined from both experiments and theory^4, 9^. Especially, the conjugation-length dependence of the 1B_u_^−^ state is not in accord with that of the dark state as observed in the emission spectra and transient absorption spectra^16–20^. Recent two-dimensional electronic spectroscopy (2DES) and high-time resolution broadband pump-probe spectroscopy measurements on Cars spheroidene (*N* = 10), rhodopin glucoside (RG) (*N* = 11) and spirilloxanthin (*N* = 13) demonstrate clearly the decay of the S_2_ state to the dark state and the energy transfer channel from the dark state to the Q_*x*_ state of Chls^6, 10, 21, 22^. From these experiments, emission energy of the dark state seems to lie a little bit (~0.1 eV) below that of the S_2_ state, irrespective of the conjugation length of Cars. Decrease of the 1B_u_^−^ energy with conjugation length is much steeper than this dark state. In addition, emission energy of the 1B_u_^−^ state might be smaller than 2 eV for Cars with *N* = 10–13 as predicted by Koyama et al.^4, 18^, according to which energy transfer to the Chls Q_*x*_ state, whose absorption energy is ~2.1 eV, cannot realize. Thus, there may exist some other, unknown excited state in the vicinity of the S_2_ state.

Another important issue is how much the dark state is involved in the Car-to-Chl energy transfer. There is disagreement in the overall Car-to-Chl energy transfer efficiency between theoretical prediction and experimental measurement, e.g., 20 vs. 50–60% for *Rhodopseudomonas (Rps.) acidophila* ^23, 24^. As the spectral overlap of the dark state emission and Q_*x*_ absorption is larger than that between the S_2_ and Q_*x*_ states, the dark state, which is not considered in previous theoretical calculations, has been supposed to account for this disagreement^6^. However, some experiments demonstrate that the amount of energy transferred from dark states to Chls is minor^4, 25, 26^.

Here, we examine the excited-state dynamics of two Cars from purple bacteria and their Car-to-Chl energy transfer by virtue of many-body Green’s function theory and Förster–Dexter theory. 2D spectrum is also simulated by a density matrix-based dynamical method to compare with experiments. We provide evidence supporting a new dark state with A_g_^+^ symmetry, which is denoted by S_*y*_, in Cars. Its absorption energy would be higher than the S_2_ state, whereas its emission energy would be lower. The S_*y*_ state is a singly excited state, in stark contrast to the well-known dark states 2A_g_^−^ and 1B_u_^−^ which are doubly excited states constituted by the two triplet excitons. The excited-state structure and the energy transfer appear to be highly sensitive to the methyl groups on the conjugated backbone of Cars.

## Results

### Excitation energy of S_*y*_

**Fig. 2:**
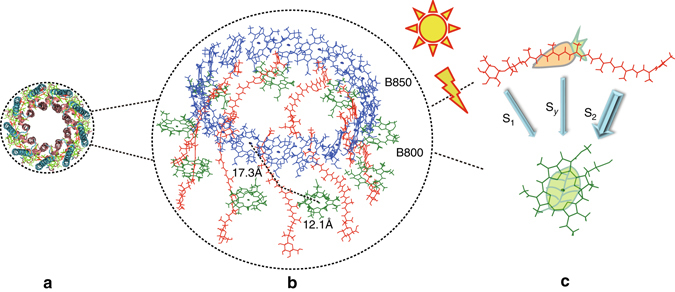
Structure of *Rps. acidophila*. **a** Top view of the structure of light-harvesting complex II in *Rps. acidophila*. **b** Enlarged view of carotenoids and chlorophylls in **a**. For a better distinction, carotenoids, the nine B800 chlorophylls, and the eighteen B850 chlorophylls are depicted in *red*, *green*, and *blue*, respectively in **b**. After these antenna pigments absorb light, carotenoids transfer energy to chlorophylls B800 and B850, mainly through the S_2_ state, partly through the S_*y*_ and S_1_ states as illustrated in **c**

**Fig. 3:**
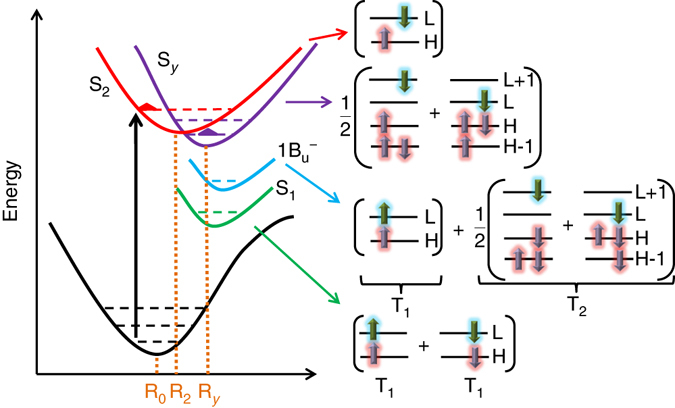
Scheme of the potential energy curves for carotenoids with *N* ≥ 9 (*left*) and the compositions of excited states (*right*). R_0_, R_2_, and R_*y*_ represent geometries at the potential minima of states S_0_, S_2_, and S_*y*_, respectively. H and L represent the highest occupied and the lowest unoccupied molecular orbitals, respectively. *Arrows* on the orbital illustrate the population and the spins of electrons in this orbital. S_2_ and S_*y*_ are singly excited states. S_1_ (formed by two T_1_) and 1B_u_^−^ (formed by T_1_ and T_2_) are doubly excited states. T_1_ and T_2_ are the lowest and the second lowest triplet exciton, respectively

**Fig. 4:**
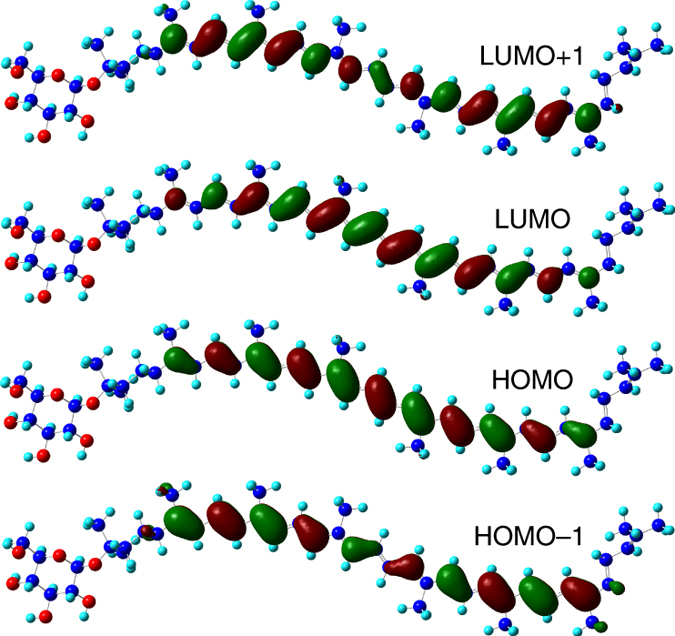
Molecular orbitals of rhodopsin glucoside. The wave function isosurfaces for the highest two occupied and the lowest two unoccupied orbitals of rhodopsin glucoside. C, O, and H atoms are shown in *blue*, *red*, and *cyan*, respectively

Extensive computational researches for the dark state in Cars have been performed using various first-principle methods^8, 14, 27–33^. However, they mainly focus on the controversial 1B_u_^−^ state. With many-body Green’s function theory, we study the excited-state dynamics of RG (*N* = 11) in *Rps. acidophila* (Fig. 2) and spirilloxanthin (*N* = 13) in *Rhodospirillum rubrum*. The calculated absorption energy of the S_2_ state for RG and spirilloxanthin are 2.65 and 2.42 eV. In experiments, the S_2_ energy is 2.48 eV for RG in methanol and 2.36 eV for spirilloxanthin in *n*-hexane^4^. In solution and the protein environment, absorption spectra of Cars redshift owing to the polarizability of the medium^4, 22, 34, 35^. If taking this into account, our calculations agree well with experiments. We also prove that putting some protein fragments near Cars has limited effects on the excitation energies of Cars (Supplementary Fig. 2). Excitation energies of the S_1_ and 1B_u_^−^ states for RG (spirilloxanthin) are calculated to be 1.87 and 2.61 eV (1.68 and 2.32 eV), respectively, employing the scheme proposed by Tavan and Schulten^36^. Above S_2_ by 0.55 eV for RG and 0.52 eV for spirilloxanthin, our results predict a new state (S_*y*_) of the A_g_^+^ symmetry. It is optically forbidden from the ground state and must be indiscernible in experimental optical absorption spectra. The S_*y*_ state is a singly excited state, with the wave function represented dominantly by the transitions HOMO−1 → LUMO and HOMO → LUMO+1 (Figs. 3, 4; Supplementary Fig. 3 and Supplementary Note 2). The high-level quantum chemistry approach EOM-CCSD can also get this state and a similar S_2_–S_*y*_ energy gap (Supplementary Table 2). The transition dipole moment of the S_*y*_ state is 0.7 and 1.6 Debye for RG and spirilloxanthin, respectively, much smaller than that of the S_2_ state (20.3 and 28.6 Debye) and comparable to the dark state measured in experiments^18^. Moreover, the S_*y*_ state remains optically forbidden when twisting the conjugated backbone of Cars from all-*trans* to *cis* configurations (Supplementary Fig. 4 and Supplementary Table 3).

### Emission energy of S_*y*_

After optical absorption, Cars first relax in the S_2_ state. From the ground-state geometry (R_0_ in Fig. 3) to the excited-state minimum geometry of the S_2_ state (R_2_ in Fig. 3), Stokes shift of the S_2_ state is 0.18 eV for both RG and spirilloxanthin. This is in accord with the shift of 0.15 eV for RG measured experimentally^4, 23^. In this process, the S_*y*_ state downshifts by 0.66 eV for RG and 0.68 eV for spirilloxanthin, respectively (Fig. 3). Now, at the potential minimum of the S_2_ state, the energy of the S_*y*_ state is just a little bit higher (0.07 eV for RG and 0.02 eV for spirilloxanthin) than the S_2_ state. If extrapolating further along the R_0_→R_2_ reaction coordinate, the energy of the S_*y*_ state falls by an additional 0.1 eV (Fig. 3). The emission energy of the S_*y*_ state (at R_*y*_ in Fig. 3) is thus lower than that of the S_2_ state (see also Supplementary Figs. 8 and 9; Supplementary Note 4). Although the crossing point between the S_2_ and S_*y*_ states is not in the R_0_→R_2_ region, nonadiabatic transition from S_2_ to S_*y*_ can happen with the aid of vibration. The S_*y*_ state may also have a role in tuning the relaxation to lower states like S_1_ (Fig. 1c). The emission energy of the S_*y*_ state for RG and spirilloxanthin is above the absorption energy of the chlorophyll Q_*x*_ state, making the Car-to-Chl energy transfer via the S_*y*_ state realizable.

**Fig. 5:**
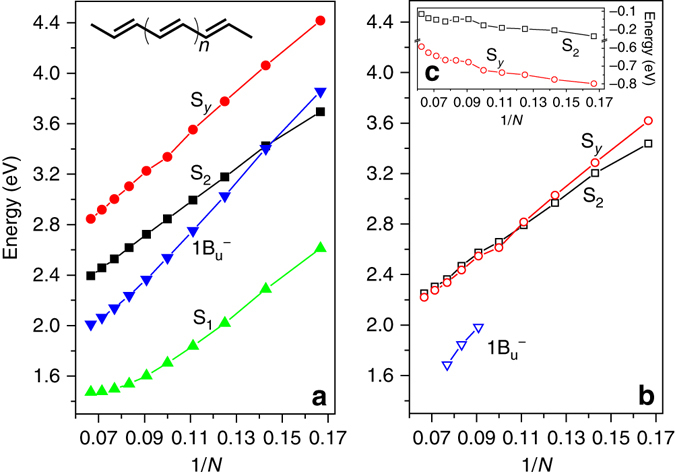
Dependence of excitation energies of polyenes on the conjugation length *N*. **a** At the ground-state geometry. **b** At the S_2_ state potential minimum geometry. **c** The energy shifts of the S_2_ and S_*y*_ states from the ground-state geometry to the S_2_ state potential minimum geometry. Energies of 1B_u_^−^ in **b** are taken from the experimental fluorescence spectra^4^

From the R_0_ to R_2_ geometries, the bond length alternation, i.e., the difference between the average lengths of C–C and C=C bonds, is reduced. As the S_2_ state is an ionic excited state, whereas the S_*y*_ state can be considered to be a covalent-like one due to its weak transition dipole moment, the electron-hole binding energy (*E*_b_) in the S_2_ state should be much more influenced by structural variation than that in the S_*y*_ state. We do find that from R_0_ to R_2_, *E*_b_ in the S_*y*_ state remains constant while that in the S_2_ state reduces by ∼0.5 eV. From R_0_ to R_2_, the gap between the unoccupied and occupied molecular orbitals (*E*_gap_) narrows by ∼0.7 eV. The excitation energy, which equals *E*_gap_−*E*_b_ in physics, thus decreases faster for the S_*y*_ state than the S_2_ state from R_0_ to R_2_ (Fig. 5c). Energies of the S_1_ and 1B_u_^−^ states are also found to exhibit much higher sensitivity on the bond length alternation than the S_2_ state^8, 36^.

### Dependence of the S_*y*_ emission energy on conjugation length

Dependence of the emission energy of the dark state on the conjugation length of Cars is a crucial factor to determine the attribution of the dark state^4^. Polyenes are ideal models to investigate this issue. We examine a series of polyenes H_3_C–(C_2_H_2_)_*N*_–CH_3_ with *N* = 6–15 (Fig. 5). Although the absorption energy of the S_*y*_ state varies faster with the conjugation length than that of the S_2_ state (Fig. 5a), the S_2_–S_*y*_ energy gap at the potential minimum of the S_2_ state remains at ∼0.03 eV for *N* ≥ 10 with the S_*y*_ below S_2_ (Fig. 5b). Taking into account the additional 0.1 eV downshift of the S_*y*_ state to its own potential minimum as discussed above, the emission energy of the S_*y*_ state is lower than that of the S_2_ state for *N* ≥ 9 and the gap between them remains at ∼ 0.1 eV. This agrees well with the transient absorption studies that the dark state lies below the S_2_ state for Cars with *N* ≥ 9^11^, and also 2DES measurements that the potential minimum of the dark state is 0.1 eV below that of the S_2_ state for *N* = 10, 11, and 13^6, 10, 21^.

### 2D spectrum of *Rps. acidophila*

**Fig. 6:**
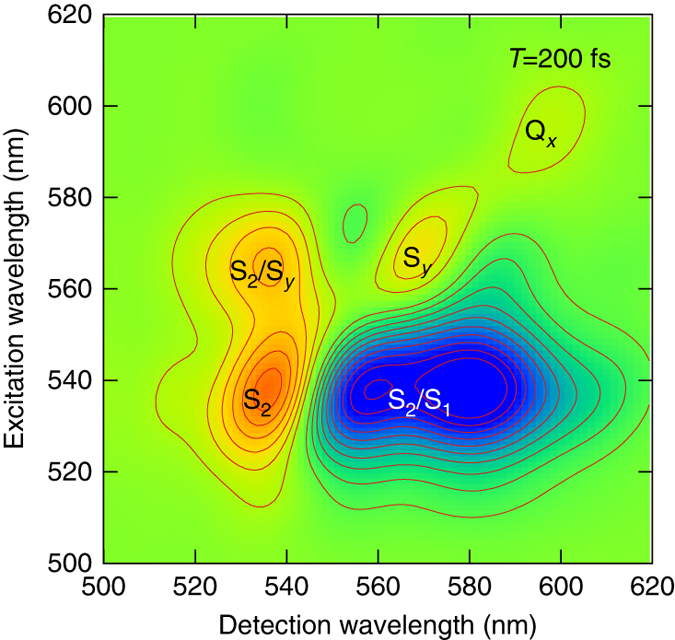
Simulated two-dimensional (2D) electronic spectrum of *Rps. acidophila* at the population time *T* = 200 fs. The diagonal peaks are symbolized by the corresponding state; the cross-peaks indicate that energy is transferred from the state represented by the *first symbol* to the acceptor state represented by the *second symbol*

In a recent study, Scholes and coworkers utilized the broadband 2DES to investigate carotenoid dark states in purple bacteria^6^. The 2DES is a four-wave mixing technique that is extremely sensitive to electronic coherence and energy relaxation dynamics^37, 38^. To verify our model, we calculate theoretical 2D spectrum of *Rps. acidophila* based on a model Hamiltonian that is consistent with our ab initio calculations. Note that the original 2D experiment is carried out using a broadband pulse that overlaps with the very red edge of the S_2_ band and the blue edge of the Q_*x*_ band in a sample of *Rps. acidophila*. The experimentally observed 2D S_2_ peak at ~535 nm (2.34 eV) is dependent of the excitation laser spectrum, and is also in good agreement with the calculated S_2_ transition energy at the S_2_ minimum. This excitation energy corresponds to a highly displaced geometry along the bond length-alternation coordinate. In this geometry, the four-state model with the S_*y*_ state energy lower than that of the S_2_ state applies, therefore, we place the S_*y*_ energy minimum at 0.1 eV below the S_2_ energy minimum in our 2D simulation, in accordance with our calculations for RG. Figure 6 shows the simulated 2D spectrum at a delay time of 200 fs, and the simulated spectrum is in agreement with the experimental spectrum^6^. Specifically, the diagonal peaks, S_2_/S_*y*_ cross-peak, and pronounced S_2_/S_1_ excited-state absorption peak that splits into two by the lower-diagonal S_2_/S_*y*_ cross-peak are all correctly reproduced in our model simulations. Therefore, our theoretical calculations are consistent with the 2D experiments.

### Influence of groups on the excitation energies of Cars

Comparing RG with the *N* = 11 polyene and spirilloxanthin with the *N* = 13 polyene, the S_*y*_−S_2_ energy gap in the Cars is the same as that in the polyene. However, the S_2_−1B_u_^−^ and S_2_−S_1_ energy gaps in the polyene are about 0.3 eV wider than those in the Cars of the same conjugation length. The structural difference between Cars and polyenes is reflected in two respects: (i) the conjugated backbone is symmetrical in polyenes but distorted in Cars, (ii) there are groups, e.g., methyl groups, on the conjugated backbone in Cars but not in polyenes. Through changing the shape of the polyene and substituting some hydrogen atoms on it by groups (Supplementary Fig. 5), we find that groups affect substantially the energy gap between the S_2_ state and the doubly excited states S_1_ and 1B_u_^−^, whereas the gap between the S_2_ and S_*y*_ states is independent of any modification to the conjugated backbone (Supplementary Table 4). This implies that energies of the S_1_ and 1B_u_^−^ states, and thus the competition between the S_2_→S_1_ decay and the Car-to-Chl energy transfer might be tuned by modifying the groups attached to the conjugated backbone.

### Energy transfer from Cars to Chls

Contribution of the dark state to the Car-to-Chl energy transfer is still an open question. It is recently proposed that the dark state-mediated energy transfer rate is of the same magnitude as the S_2_-mediated one^6, 21^. This is in contradiction with previous work^4, 25, 26^. The nonadiabatic transition from the S_2_ to S_*y*_ states is always present for longer Cars according to discussions in the previous sections. The S_*y*_ state must be involved in the Car-to-Chl energy transfer. We thus further investigate the energy transfer capability of the S_*y*_ state, which is important for understanding the energy transfer mechanism in photosynthesis and resolving the controversies in this respect.

**Fig. 7:**
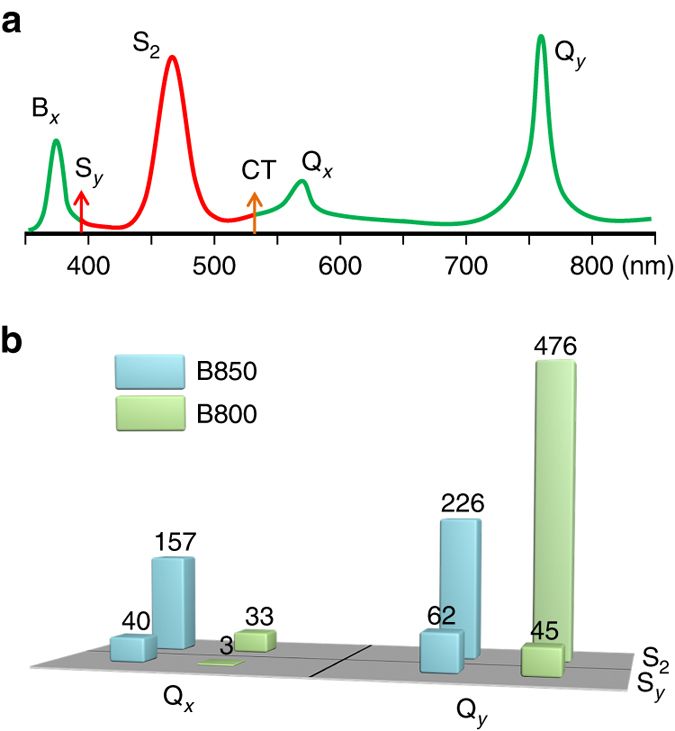
Absorption spectrum and electronic coupling strength for a carotenoid–chlorophyll complex. **a** Calculated absorption spectrum of the rhodopin glucoside-B800 complex as linked by the *dashed line* in Fig. 2b. CT denotes the charge-transfer state. Oscillator strengths of the S_*y*_ and CT states are very weak. Here *arrows* are used to indicate their positions. **b** Electronic coupling strengths (in cm^−1^) for the energy transfer passways from the S_2_ and S_*y*_ states to the Q_*x*_ and Q_*y*_ states in the rhodopin glucoside-B850 and rhodopin glucoside-B800 pairs as linked by *dashed lines* in Fig. 2b. Distances between the centers of corresponding rhodopin glucoside and B850/B800 molecules are given in Fig. 2b

We calculate the energy transfer in the RG-B850 and RG-B800 pairs in *Rps. acidophila* as linked by *dashed lines* in Fig. 2b. Energy flow in these two kinds of pairs has been supposed to dominate the Car-to-Chl energy transfer^24^. Figure 7a shows the calculated absorption spectrum of the RG-B800 pairs, which is comparable to the experimental spectrum of LH2 complex in *Rps. acidophila* strain 10050^3, 6^. Positions of the Q_*x*_ and Q_*y*_ peaks deviate from the experimental ones by <0.1 eV. A charge-transfer state, with the electron excited from the Car to the Chl, appears between the S_2_ and Q_*x*_ states, which can result in the formation of Car radicals as detected in experiments^4^. The rate of energy transfer is evaluated via *k* = (2*π*/ℏ)|*V*_DA_|^2^ *J*_DA_, where *V*_DA_ and *J*_DA_ are the electronic coupling strength and the spectral overlap between donor (D) and acceptor (A) transitions, respectively. *V*_DA_ is calculated within the framework of many-body Green’s function theory.

We use the spectral overlap data from Krueger et al.^24^ and Ostroumov et al.^21^, where *J*_DA_(S_2_−Q_*x*_) = 13*J*_DA_(S_2_−Q_*y*_) and *J*_DA_(S_2_−Q_*x*_) = *J*_DA_(S_*y*_−Q_*x*_). Figure 7b compares the electronic coupling strength for each energy transfer passway from Car to Chl. We find that the energy transfer rate of the S_2_-B800 Q_*y*_ channel is much higher than expected theoretically before^24^ and can reach 70% of that of the S_2_-B850 Q_*x*_ channel which has been considered to be the dominating channel. This branching ratio is in good agreement with the experiment^39^, supporting the experimental observations that both Q_*x*_ and Q_*y*_ have the role of energy acceptors and the amount of energy transferred to B800 is comparable to that to B850^4, 39–42^. The S_*y*_-mediated energy transfer is predominated by the S_*y*_-B850 Q_*x*_ channel. The overall S_*y*_-mediated energy transfer rate is one order of magnitude smaller than that of the S_2_-mediated one. Thus, although the dark state participates in the energy transfer process, the Car-to-Chl energy transfer is still governed by the S_2_ state (see Fig. 2c for the energy flow).

## Discussion

Ever since the discovery of the intermediate dark state between the S_2_ and S_1_ states in Cars by Cerullo et al. in 2002, its role in photosynthesis becomes increasingly emphasized^5^. On the basis of the conventional theoretical model for the electronic structures of polyenes developed by Tavan and Schulten^36^, the 1B_u_^−^ state has long been regarded as the candidate for the dark state. The strongly dependence of the S_2_–1B_u_^−^ energy gap on the conjugation length make this assignment questionable^4, 9^. The S_*y*_ state of the A_g_^+^ symmetry has a more moderate conjugation length dependence than the 1B_u_^−^ state. More importantly, variation of the S_*y*_ emission energy with respect to the conjugation length is parallel to the S_2_ state, and their energy gap is kept at a small value (~ 0.1 eV) for Cars with *N* > 9. This is consistent with the experimental findings, such as those in 2DES, that the emission energy gap between the dark state and the S_2_ state is about 300 cm^−1^ and independent of the conjugation length^4, 6, 10, 21^. This small energy gap ensures the extremely fast (~10 fs) internal conversion from the S_2_ state to the dark state as observed experimentally^4, 5, 11^. The agreement between our theoretical 2D spectrum (Fig. 6) and the experimental data lends support to our model with a new A_g_^+^ state below S_2_ at a large bond-length alternation. Interestingly, the experimental 2D study resolves the diagonal peak due to the dark state. Furthermore, based on ultrafast-transient absorption and transient-grating experiments, some groups have suggested that the dark-state is due to a double-minima structure on the S_2_ potential energy surface, not a distinct electronic state^43, 44^. This double-minima model hypothesizes that the lowest one of the S_2_ potential surface minima exists at the conformation where the carotenoid is twisted from the all-*trans* isomer by ~90° with respect to one C=C bond of the conjugated polyene backbone. This may be true for molecules with a short conjugated backbone such as the protonated Schiff bases^45^, which is also demonstrated in our previous theoretical work^46^. Nevertheless, this does not seem to be the case for molecules with a long conjugated backbone (Supplementary Fig. 10). The new state described in the present article, with strong energy shift along the bond length alternation coordinate that crossovers in energy with the S_2_ state (Fig. 3), does exhibit a double-well like feature that may explain the spectral shifts observed in the recent experiments. Noticeably, the theoretical 2D spectrum based on our model correctly describes the S_2_/S_*y*_ cross-peak above the diagonal in the experiment, whereas in the double-minima model one should expect to see extensive spectral diffusion and an elongated S_2_ peak along the detection wavelength (below the diagonal), which is inconsistent with the 2D experimental data. In addition, the clear diagonal S_*y*_ peak is also not explained by the double-minima model. On the basis of the above analysis, a carotenoid photophysics four-state model is given in Fig. 1c, which involves the S_1_, S_*y*_, and S_2_ excited states.

One important motive to study the dark state in experiments is to solve the obvious distinction in the estimated Car-to-Chl energy transfer efficiency between previous theoretical calculations (20%) and experimental measurements (50–60%)^6, 21, 24^. Above we have illustrated that the portion of energy transferred via the S_*y*_ state is minor based on the assumption *J*_DA_(S_2_−Q_*x*_) = *J*_DA_(S_*y*_−Q_*x*_) proposed by Ostroumov et al.^21, 27^. Even if *J*_DA_(S_*y*_−Q_*x*_) > *J*_DA_(S_2_−Q_*x*_), considering the smaller energy gap between the S_*y*_ and Q_*x*_ states than that between the S_2_ and Q_*x*_ states, the contribution of the S_*y*_ state cannot fill the gap between theoretical calculations and experiments. The electronic coupling strengths we calculate by many-body Green’s function theory are 1.5 times stronger than those from previous theoretical calculations by the transition density-cube approach^24^. We suggest that the disagreement in energy transfer efficiency between previous theoretical work and experiments may be due not only to the absence of the dark state in the theoretical model, but also to the underestimation of electronic coupling strengths in previous calculations.

In conclusion, our study provides evidence for a new state S_*y*_ of the A_g_^+^ symmetry in Cars, thus contributing to a better understanding of carotenoid excited states. Future experiments would be required to test the accuracy of our calculations.

## Methods

### Ground-state geometry

Density-functional theory (DFT) with the Coulomb-attenuating method variant of the Becke 3-parameter-Lee-Yang-Parr (CAM-B3LYP) exchange-correlation functional^47^ is used to optimize geometries of Cars and Chls by the Gaussian 09 program^48^. CAM-B3LYP has been shown to give more reasonable structures than other functionals^14, 49^.

### Excitation energy

Many-body Green’s function theory, which includes the combination of GW method and Bethe–Salpeter equation (BSE)^50, 51^, is applied to compute the excitation energies with a Gaussian orbital based GW-BSE package^52, 53^. Calculations are performed at the level of full BSE, i.e., considering the mixing between resonant and antiresonant transitions, as Tamm–Dancoff approximation can cause large errors for organic molecules^54–56^. This scheme has been applied for electronic excitations in many organic systems^54, 57–59^. The S_1_ and 1B_u_^−^ states bear doubly excited character, involving two coupled triplet excitations. Their excitation energies cannot be obtained from BSE directly as BSE can only deal with one electron–hole pair excitation. Here, we estimate their energies according to Tavan and Schulten’s theory^36^, i.e., *E*(S_1_) = 2*E*(T_1_) and $E\left( {1{\rm{B}}_{\rm{u}}^ - } \right) = E\left( {{{\rm{T}}_1}} \right) + E\left( {{{\rm{T}}_2}} \right)$_,_ where T_1_ and T_2_ are the lowest two triplet states of Cars and are computed via GW-BSE. This approach possesses high accuracy as proved by Tavan and Schulten. In the Supplementary Note 1, Supplementary Fig. 1, and Supplementary Table 1, a detailed discussion on the accuracy of our strategy to predict the S_1_ and 1B_u_^−^ energies is presented.

### Potential minimum at the excited state

Equilibrium structure of Cars in the S_2_ state, which originates from the HOMO → LUMO transition (point R_2_ in Fig. 3) is optimized by the constrained density-functional theory (CDFT) where occupations in HOMO and LUMO are fixed at 1 during structural relaxation^60^. As the S_*y*_ state is composed predominantly by transitions HOMO-1 → LUMO and HOMO → LUMO+1 with equal weight, its potential minimum cannot be predicted by structural optimization on its own energy surface using CDFT. We locate the potential minimum of the S_*y*_ state (point R_*y*_ in Fig. 3) approximately by extrapolating along the R_0_ → R_2_ reaction coordinate of the S_2_ state. We validate the reasonability of the S_*y*_ potential minimum predicted by this scheme through relaxing Cars in the S_*y*_ state with excited-state forces from BSE. BSE forces are computed by the approach proposed by Ismail-Beigi and Louie with the exception that we use finite difference method to evaluate derivatives of single-particle energies and wave functions with respect to nuclear positions^61, 62^. This technique to compute BSE forces has been well tested to give consistent results with those from Ismail-Beigi and Louie on the excited-state structures of CO and NH_3_ molecules^61^ (see Supplementary Note 3, Supplementary Table 5, and Supplementary Figs. 6 and 7 for details of the theory and test of the accuracy).

### Theoretical 2D spectrum

We utilize a density matrix-based dynamical method to simulate 2D spectrum of *Rps. acidophila*. This method accounts for full density-matrix dynamics and bath memory effects in the simulated 2D spectrum, and details of the theory are described in refs ^63, 64^. The model adopted for the *Rps. acidophila* system consists of five carotenoid states (S_0_, S_1_, S_*y*_, S_2_, and S_*n*_) and one chlorophyll state (Q_*x*_). The transition energies are set at 16,900, 17,810, 18,620, and 17,500 cm^−1^ for Q_*x*_, S_*y*_, S_2_, and S_1_ → S_*n*_, respectively. This model places the S_*y*_ energy at 0.1 eV below the S_2_ state to describe carotenoid electronic states at a geometry highly displaced along the bond length-alternation coordinate, in accordance with our calculations for RG. The dynamics are described by a Lindblad master equation including five population relaxation terms: ${\tau _{{{\rm{S}}_2} \to {{\rm{S}}_y}}}$ =  0.3 ps, ${\tau _{{{\rm{S}}_y} \to {{\rm{S}}_1}}}$ = 1 ps, ${\tau _{{{\rm{S}}_y} \to {{\rm{Q}}_x}}}$=2 ps, ${\tau _{{{\rm{S}}_1} \to {{\rm{S}}_0}}}$=3 ps, ${\tau _{{{\rm{Q}}_x} \to {{\rm{S}}_0}}}$=1 ps. The line broadenings are described by Gaussian static disorders with *σ* = 450 cm^−1^, and couplings to a super-Ohmic bath with a spectral density $J\left( \omega \right) = {\gamma _0}\frac{{{\omega ^3}}}{{\omega _{\rm{c}}^2}} {\rm {e}}^{ - \omega /{\omega _{\rm{c}}}}$. The coupling strength *γ*_0_ and cut-off *ω*_c_ are set at 0.6 and 850 cm^−1^, respectively. Note that in this work, we aim to demonstrate that our model produces 2D spectrum that is consistent with experimental results, therefore we do not perform full fittings to the experimental spectra, and the bath and dynamical parameters used in our model are only set tentatively. Additional calculations that provide nonadiabatic couplings and explore the potential surfaces more completely are required to fully describe the experimental 2D data.

### Energy transfer

The electronic energy transfer rate, *k*, is calculated via *k* = (2*π*/ℏ)|*V*_DA_|^2^ *J*_DA_ according to the Förster–Dexter theory. The electronic coupling strength *V*_DA_ is computed within the framework of many-body Green’s function theory via

1 $${V_{{\rm{DA}}}} = X_{\rm{D}}^T\left( {K_{{\rm{DA}}}^x + K_{{\rm{DA}}}^d} \right){X_{\rm{A}}} - {\omega _0}X_{\rm{D}}^T{S_{{\rm{DA}}}}{X_{\rm{A}}}$$

where *X*_D/A_ is the BSE exciton wave function for donor (D) and acceptor (A), ω_0_ the energy transferred, *S*_DA_ the overlap matrix between donor and acceptor orbitals. *K* ^*x*^ and *K* ^*d*^ are the exchange and direct terms of the BSE electron–hole interaction kernel. Electronic coupling arising from these two terms resemble the Förster and Dexter coupling, respectively^65^. *V*_DA_ computed by Eq. (1) consists excellently with that by the high-level quantum chemistry approach CASSCF^66^.

### Data availability

All data supporting the findings of this study are available from the corresponding author on request.

## Electronic supplementary material

Supplementary Information

Peer Review File
