# Supplementary material for: A new energy transfer channel from carotenoids to chlorophylls in purple bacteria
Source: Nat Commun. 2017 Jul 10;8:71. doi: 10.1038/s41467-017-00120-7 (PMC5504074; doi:10.1038/s41467-017-00120-7)
Supplement: Supplementary file 1 — Supplementary Information [file 41467_2017_120_MOESM1_ESM.pdf]

File name: Supplementary Information
Description: Supplementary Figures, Supplementary Tables, Supplementary Notes and Supplementary References

File name: Peer Review File
Description:

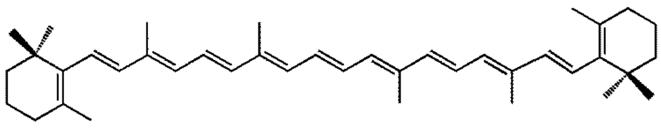

Supplementary Figure 1. Structure of β-carotene.

Method	$2A_g^-$	$1B_u^-$	$1B_u^+$
GW-BSE	1.99	2.82	2.64
DFT/MRCI (ref. 1)	2.01	2.65	2.42

Supplementary Table 1. Comparison of the excited-state absorption energies (eV) of β-carotene from different approaches.

Supplementary Note 1:

Test of GW-BSE method for the $2A_g^-$ and $1B_u^-$ state

The $2A_g^-$ and $1B_u^-$ states are formed by two coupled triplet excitations. Their excitation energies cannot be obtained from BSE directly since BSE can only deal with one electron-hole pair excitation. In the work by Tavan and Schulten[2], they find that energies of the $2A_g^-$ and $1B_u^-$ states for polyenes can be approximated at a high precision by the sum of two triplet energies, i.e. $E(2A_g^-) \approx 2E(T_1)$ and $E(1B_u^-) \approx E(T_1) + E(T_2)$ where T_1 and T_2 are the lowest two triplet states, based on their calculations using the multiple-reference double excitation configuration-interaction method (MRD-CI). For example, energies of the $2A_g^-$ and $1B_u^-$ states for the N=10 polyene are 3.40 and 4.23 eV from exact MRD-CI calculations, while sums of the exact MRD-CI energies of the associated triplets for the $2A_g^-$ and $1B_u^-$ states are 3.51 and 4.32 eV (see Table III in Ref.2). Accuracy of this approximation for the absolute energies of the two states is 0.1 eV, while accuracy for the energy difference between these two states reaches 0.02 eV. Therefore this kind of approximation is a suitable approach to predict the energies of the $2A_g^-$ and $1B_u^-$ states. To further verify the reliability of this approximation and the ability of BSE to study these double excitations based on this approximation, we evaluate the excited states of β-carotene (N = 11) (Figure 1) whose energies of the $2A_g^-$ and $1B_u^-$ states from the high-level configuration-interaction method are available. Table 1 compares absorption energies of the $2A_g^-$, $1B_u^-$ and $1B_u^+$ states from our BSE and those from

DFT/MRCI by Ostroumov *et al*[2]. Results from BSE compare well with those from DFT/MRCI. Especially the energy difference between $1B_u^-$ and $1B_u^+$ states, which is mostly concerned in this work, from these two methods is very close (within 0.05 eV).

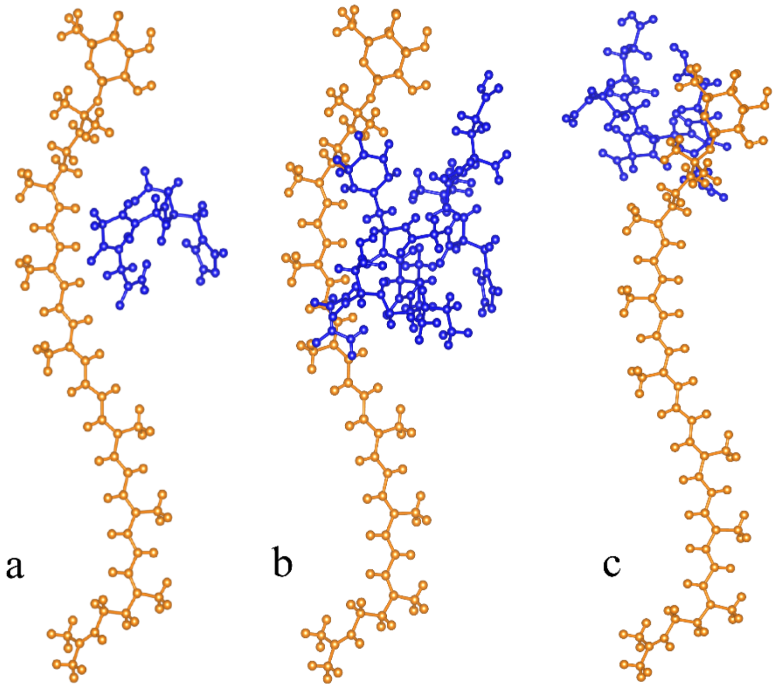

d

States	Isolated RG	a	b	c
S_y	3.20	3.21	3.20	3.19
S_2	2.65	2.59	2.63	2.63

Supplementary Figure 2. Influence of protein environment on the excitation energies of Cars (a-c). Three kinds of structures for rhodopin glucoside (RG) (N=11) with some protein fragments (in blue). Configuration of RG is optimized by DFT using the CAM-B3LYP functional. Configuration of the protein fragments and their relative position with respect to RG are taken from the Protein Data Bank (2FKW)[3]. To find how much the protein environment affects excitation energies of Cars, we put some representative protein fragments in the vicinity of RG as shown. These protein fragments contain 44, 112 and 74 atoms respectively for structures a, b and c. The shortest distances between atoms in these fragments and atoms in RG are ~3.8 Å. The influence of distant part of protein on the excited states should be much less than these close fragments. In these calculations geometry of RG is fixed at that optimized by DFT with the CAM-B3LYP functional, atoms of the protein fragments are fixed at their positions extracted from the Protein Data Bank. **(d)** Excitation energies (eV) of the S_2 and S_y states for isolated RG without protein environment and those for RG with protein

fragments nearby (structures a, b and c). It can be seen that effect of the protein fragments on excitation energies of the S_2 and S_y states is limited. Therefore, we believe that the protein environment would not substantially alter the conclusion of our work.

States	Composition	CAM-B3LYP(BSE)	CAM-B3LYP	B3LYP	BLYP
$1A_g^+$	(H−1→L)−(H→L+1)	3.84	5.05	4.9	4.79
S_y (nA_g^+)	(H−1→L)+(H→L+1)	3.23	4.70	4.32	4.02
S_2 ($1B_u^+$)	H→L	2.67	4.06	3.81	3.62

Supplementary Table 2. Composition and absorption energies (eV) of the lowest three singly excited states in BSE and their correspondences in EOM-CCSD for the N=11 polyene (Figure 3). Only the dominant contributions to the three states are listed in the 2nd column. H and L denote HOMO and LUMO. The 3rd column is for BSE calculations on the CAM-B3LYP optimized structure. The 4th to 6th columns are for EOM-CCSD calculations on the CAM-B3LYP, B3LYP and BLYP optimized structures.

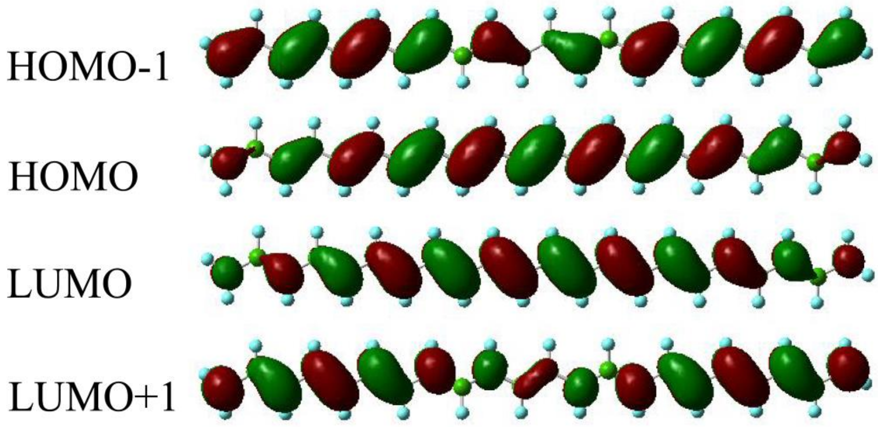

Supplementary Figure 3. Molecular orbitals for the N=11 polyene.

Supplementary Note 2:

EOM-CCSD study on the N=11 polyene

In this section we give a comparison between BSE method and EOM-CCSD for the N=11 polyene. BSE is a theory to study excitation of one electron. Here, we only discuss the singlet excited states which involve excitation of only one electron. Doubly excited states which are composed by

two triplet excitations and involve excitation of two electrons, such as $2A_g^-$ and $1B_u^-$, are not considered here. Ground-state structure optimization and EOM-CCSD calculations are performed by the Gaussian 09 code[4]. From Table 2, the lowest $1B_u^+$ state comes from the HOMO→LUMO transition. The second lowest state is the S_y state which is formed dominantly by in-phase transitions HOMO→LUMO+1 and HOMO-1→LUMO in BSE. Although S_y is formed by two transitions, it is a singly excitation in nature which is different from the doubly excited states $2A_g^-$ and $1B_u^-$. In BSE, wave function (Ψ) of the exciton (electron-hole pair) for certain excited state (*S*) is expanded by the products of occupied and virtual molecular orbitals, i.e.

$$\Psi_S(\mathbf{r}_h, \mathbf{r}_e) = \sum_i^{occupied} \sum_a^{virtual} A_{ia}^S \psi_i(\mathbf{r}_h)\, \psi_a(\mathbf{r}_e)$$

where ψ_i and ψ_a are the occupied and virtual molecular orbitals for the ground state before excitation, $\mathbf{r}_h$ and $\mathbf{r}_e$ are coordinates of the hole and excited electron (after excitation the excited electron occupies the original virtual molecular orbitals, while the hole takes the original occupied ones), A_{ia}^S is the coefficient for the contribution of one product $\psi_i(\mathbf{r}_h)\psi_a(\mathbf{r}_e)$ to the whole exciton wave function. $|A_{ia}^S|^2$ is the weight of transition $i \rightarrow a$ to the whole excitation. Wave function for the exciton in the S_y state from BSE is

$$\Psi_{S_y}(\mathbf{r}_h, \mathbf{r}_e) = 0.66\psi_{\mathrm{H}-1}(\mathbf{r}_h)\psi_{\mathrm{L}}(\mathbf{r}_e) + 0.63\psi_{\mathrm{H}}(\mathbf{r}_h)\psi_{\mathrm{L}+1}(\mathbf{r}_e) \\ + 0.25\psi_{\mathrm{H}-2}(\mathbf{r}_h)\psi_{\mathrm{L}+1}(\mathbf{r}_e) - 0.24\psi_{\mathrm{H}-1}(\mathbf{r}_h)\psi_{\mathrm{L}+2}(\mathbf{r}_e) + \text{other minor contributions}$$

Wave function of the third lowest state in BSE is

$$\Psi_{1\mathrm{A}_\mathrm{g}^+}(\mathbf{r}_h, \mathbf{r}_e) = 0.67\psi_{\mathrm{H}-1}(\mathbf{r}_h)\psi_{\mathrm{L}}(\mathbf{r}_e) - 0.70\psi_{\mathrm{H}}(\mathbf{r}_h)\psi_{\mathrm{L}+1}(\mathbf{r}_e) \\ + 0.1\psi_{\mathrm{H}-3}(\mathbf{r}_h)\psi_{\mathrm{L}}(\mathbf{r}_e) - 0.1\psi_{\mathrm{H}-1}(\mathbf{r}_h)\psi_{\mathrm{L}+2}(\mathbf{r}_e) + \text{other minor contributions}$$

Composition of this state is similar to the S_y state except that the contributions HOMO→LUMO+1 and HOMO-1→LUMO are antiphase in this state but in-phase in S_y. We assign this state to the $1A_g^+$ state as observed in experiments[5-10] based on following analyses:

(1) In experiments, the absorption energy of the $1A_g^+$ state is above the S_2 state by 0.94 eV for lycopene (N=11)[6, 11]. In Table 2, this state is above the S_2 state by ~1 eV for both BSE and EOM-CCSD.

(2) Experiments observe that $1A_g^+$ state is optically forbidden for the all-*trans* configuration of Cars. When Cars is twisted to the *cis* configuration, oscillator strength of the $1A_g^+$ state increases, at

the same time the S_2 absorption blueshifts[7, 8, 12]. We also calculate excited states of the N=11 polyene in the *cis* configurations, as shown in Figure 4 and Table 3 for the structures and results. It is clearly seen that oscillator strength of the third lowest state is in the order, all-*trans* < 5-*cis* < 7-*cis* < 11-*cis*, the S_2 energy blueshifts by 7 nm. These are in accordance with properties of $1A_g^+$ in experiments.

Based on the agreement between the third lowest state and the $1A_g^+$ state in both the energy and variation of oscillator strength, it is reasonable to attribute the third lowest state in BSE to $1A_g^+$.

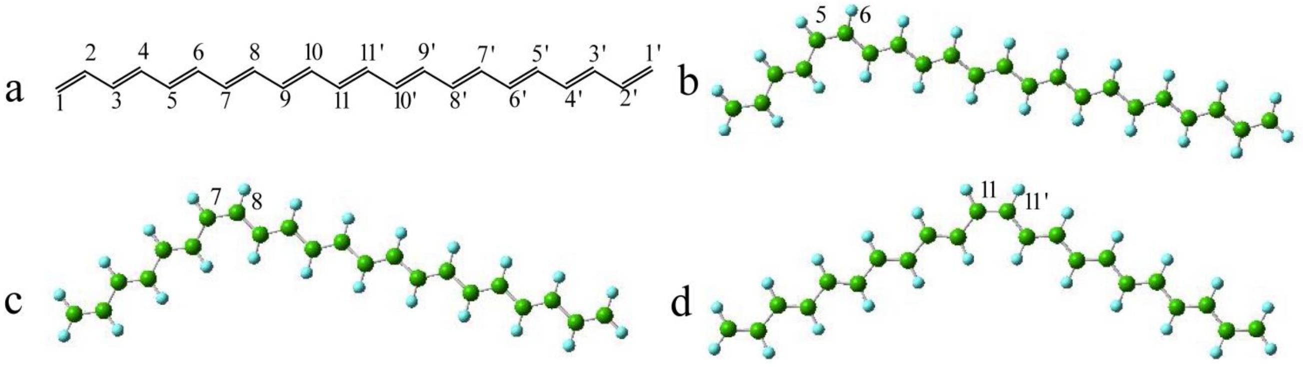

Supplementary Figure 4. The configurations of isomeric N=11 polyene optimized by DFT using the CAM-B3LYP functional: **(a)** all-*trans*; **(b)** 5-*cis*; **(c)** 7-*cis*; **(d)** 11-*cis*.

States	all-*trans*	5-*cis*	7-*cis*	11-*cis*
$1A_g^+$	3.84 (0.00)	3.85 (0.05)	3.83 (0.16)	3.85 (0.34)
S_y (nA_g^+)	3.23 (0.00)	3.28 (0.00)	3.28 (0.00)	3.26 (0.00)
S_2 ($1B_u^+$)	2.67 (2.61)	2.70 (2.48)	2.71 (2.36)	2.71 (2.23)

Supplementary Table 3. Absorption energies (eV) of the lowest three singly excited states of the N=11 polyene in the all-*trans*, 5-*cis*, 7-*cis* and 11-*cis* configurations (Figure 4). Oscillator strength is given in the parentheses.

The S_y state is also of A_g symmetry from Gaussian 09 calculations. It is likely another A_g^+ state that has never been observed before.

In EOM-CCSD, wave function of the S_y state is

$$
\begin{aligned}
\Psi_{S_y}^{\text{EOM-CCSD}} = \;& 0.55(\text{HOMO}-1 \rightarrow \text{LUMO}) + 0.48(\text{HOMO} \rightarrow \text{LUMO}+1) \\
+ \;& 0.27(\text{HOMO}-2 \rightarrow \text{LUMO}+1) - 0.25(\text{HOMO}-1 \rightarrow \text{LUMO}+2) \\
- \;& 0.23(\text{HOMO}^2 \rightarrow \text{LUMO}^2) + \text{other minor contributions}
\end{aligned}
$$

The same as BSE, transitions HOMO→LUMO+1 and HOMO-1→LUMO are the key components. Although there is one double-excitation contribution $\text{HOMO}^2 \rightarrow \text{LUMO}^2$, its weight is so small that it cannot influence the singly-excitation character of the S_y state.

EOM-CCSD has been used to study excited states in Cars[9, 13-16]. Sometimes it overestimates excitation energies by a large amount. For example, it overestimates the S_2 energy of peridinin by more than 1 eV in Ref.13. In our calculations, EOM-CCSD also overestimates the S_2 energy of the N=11 polyene by more than 1 eV (Table 2). However, the energy difference between the states S_2, S_y and $1A_g^+$ from EOM-CCSD is close to that from BSE. Thus, our BSE prediction of the S_y state is reliable.

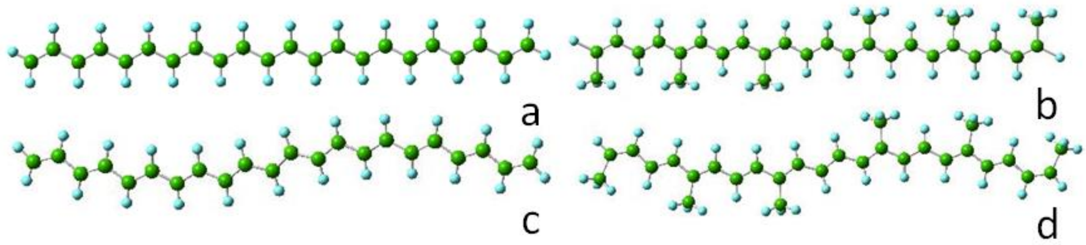

Supplementary Figure 5. Structures of the N=11 polyenes. **a)** An optimized $C_{22}H_{24}$. **b)** Six H atoms in a) are replaced by $-CH_3$ groups with position of the conjugate backbone fixed while position of the $-CH_3$ groups and H atoms optimized. **d)** is the structure after all the atoms in b) are relaxed. **c)** Six $-CH_3$ groups in d) are replaced by H atoms with position of the conjugate backbone fixed while position of the H atoms optimized.

Structure	$2A_g^-$	$1B_u^-$	$1B_u^+$	S_y
a	1.34	2.16	2.67	3.23
b	1.49	2.28	2.54	3.10
c	1.29	2.13	2.65	3.20
d	1.53	2.32	2.57	3.11

Supplementary Table 4. Absorption energies (eV) of the excited states for structures in Figure 5.

CO	Ground state ($X^1\Sigma^+$)		NH$_3$	Ground state ($\widetilde{X}^1A_1$)		
	R_e (Å)	IP (eV)		R_e (Å)	$\theta(^\circ)$	IP (eV)
LDA	1.13	8.75	LDA	1.02	109.0	5.7
GW	···	13.81	GW	···	···	10.3
Expt.	1.128	14.01	Expt.	1.01	106.7	10.1
	Excited state ($A^1\Pi$)			Excited state ($\widetilde{A}^1A_2''$)		
	R_e (Å)	T_e (eV)		R_e (Å)	θ ($^\circ$)	T_e (eV)
EOM-CCSD	1.22	7.91	CASSCF	1.06	120	5.49
GW-BSE	1.26	8.36	GW-BSE	1.09	120	6.15
Expt.	1.24	8.07	Expt.	1.08	120	5.7

Supplementary Table 5. Ground-state and excited-state data for CO and NH$_3$. R_e: equilibrium bond length; θ: H-N-H angle; T_e: minimum-to-minimum energy for $X^1\sum^+ \rightarrow A^1\prod$ for CO and $\tilde{X}^1A_1 \rightarrow \tilde{A}^1A_2''$ for NH$_3$; IP: ionization potential. The LDA and GW data for the ground state and GW-BSE data for the excited state are from our calculations, EOM-CCSD from Ref.17, CASSCF from Ref.18, experiment data (Expt.) from Ref.19 for CO and Ref.20 for NH$_3$.

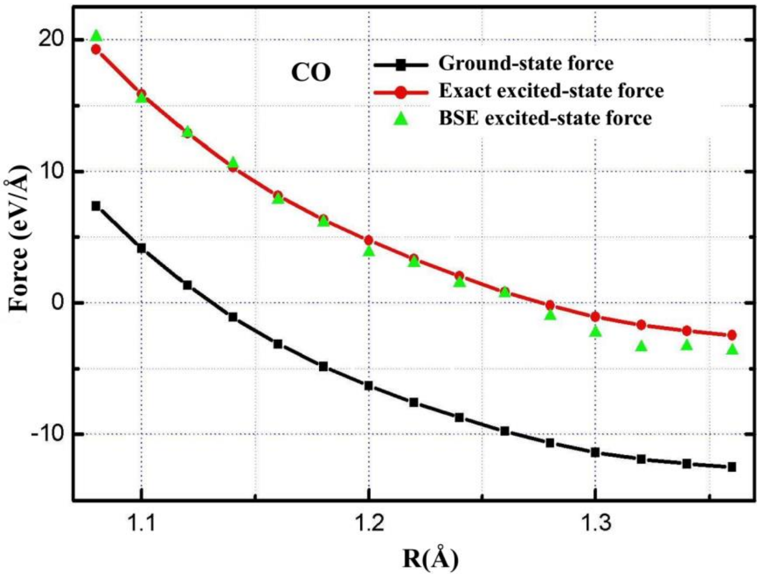

Supplementary Figure 6. Ground-state and $A^1\prod$ excited-state forces for CO as the function of C-O bond length. "Exact excited-state force" is calculated by the finite difference method applied on the GW-BSE excitation energy plus the Hellmann-Feynman force of the ground state. "BSE excited-state force" is calculated by the approach described below in this section plus the Hellmann-Feynman force.

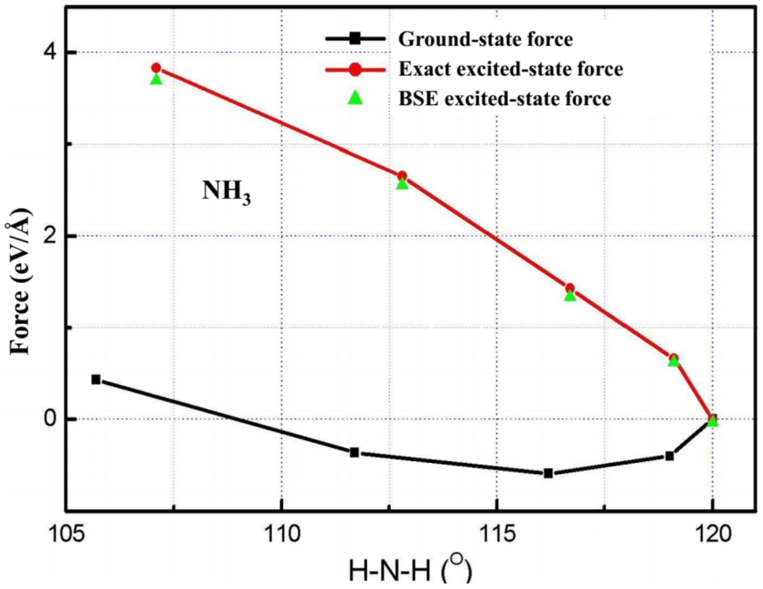

Supplementary Figure 7. Ground-state and $\tilde{A}^1A_2''$ excited-state forces for NH_3 as the function of H-N-H bond angle. "Exact excited-state force" is calculated by the finite difference method applied on the GW-BSE excitation energy plus the Hellmann-Feynman force of the ground state. "BSE excited-state force" is calculated by the approach described below in this section plus the Hellmann-Feynman force.

Supplementary Note 3:

Details about the excited-state force calculated by GW-BSE

Total energy of the excited state (E_s) in BSE is the sum of the ground-state total energy (E_{DFT}) and the excitation energy for this state (Ω_s)

$$E_s = E_{DFT} + \Omega_s$$

The force in this excited state is composed by the Hellmann-Feynman force in the ground state and the derivatives of the excitation energy Ω_s versus the nuclear coordinate R

$$F_R^S = -\frac{\partial E_S}{\partial R} = -\left[\frac{\partial E_{DFT}}{\partial R} + \frac{\partial \Omega_S}{\partial R}\right]$$

The BSE eigenvalue equation is

$$\sum_{v'c'} H^{BSE}_{vc,v'c'} A^S_{v'c'} = \Omega_S A^S_{vc}$$

with the BSE Hamiltonian (H^{BSE}) taking the form

$$H^{BSE}_{vc,v'c'} = \left(E^{QP}_c - E^{QP}_v\right)\delta_{cc'}\delta_{vv'} + K^{BSE}_{vc,v'c'}$$

where E^{QP}_c and E^{QP}_v are the quasiparticle (QP) energies for the conduction band c (or virtual orbital) and the valance band v (or occupied orbital) evaluated by the GW method, K^{BSE} is the electron-hole interaction kernel[21, 22]. Since the wave function coefficients fulfill the normalization condition

$$\sum_{vc} |A^S_{vc}|^2 = 1$$

the derivative of Ω_s versus the nuclear coordinate R ($\partial_R \Omega_S$) can be calculated by

$$\partial_R \Omega_S = \sum_{vc,v'c'} A^{S*}_{vc} A^S_{v'c'} \partial_R H^{BSE}_{vc,v'c'}$$

where

$$\partial_R H^{BSE}_{vc,v'c'} = \left(\partial_R E^{QP}_c - \partial_R E^{QP}_v\right)\delta_{cc'}\delta_{vv'} + \partial_R K_{vc,v'c'}$$

Based on the theory for BSE excited-state force proposed by Ismail-Beigi and Louie[23], the derivatives of the electron-hole interaction kernel can be expressed by

$$\partial_R K_{vc,v'c'} = \sum_j \left[P^R_{jv} K_{jc,v'c'} + P^{R*}_{jc} K_{vj,v'c'} + P^{R*}_{jv'} K_{vc,jc'} + P^R_{jc'} K_{vc,v'j}\right]$$

where $P^R_{ji} = \langle j|\{\partial_R|i\}$. Here the completeness condition over molecular orbitals $\sum_j |j\rangle\langle j| = 1$ is used. In their work, Ismail-Beigi and Louie employ density functional perturbation theory and first order perturbation theory to obtain $\partial_R E^{QP}_i$ $(i = v, c)$ and P^R_{ji} which are the only extra computational burdens beyond standard GW-BSE. In our work, we apply the finite difference method to calculate $\partial_R E^{QP}_i$ and P^R_{ji} instead. The off-diagonal matrix elements of the quasiparticle Hamiltonian H^{QP} in the GW procedure are also taken into account in order to achieve accurate BSE energies and reasonable excited-state geometries as discussed by Ismail-Beigi and Louie. Table 5 lists the relaxed excited-state structures and energies of CO in the $A^1\prod$ state and NH_3 in the $\tilde{A}^1A''_2$ state by our method. The calculated bond length, bond angle and $0 \rightarrow 0$ transition energy agree with both experiments and high-level quantum chemistry approaches, and are also consistent with results from Ismail-Beigi and Louie[23]. The bond length of CO is elongated and NH_3 turns to be a planer structure in the excited state. Figure 6 and 7 compare the BSE forces by our approach and the exact excited-state forces which are evaluated by the finite difference scheme directly over the BSE total energy $E_s = E_{DFT} +$

Ω_s in the excited states. It can be seen from these two figures, together with Table 5, that our approaches and codes could predict reliable geometries in the excited states.

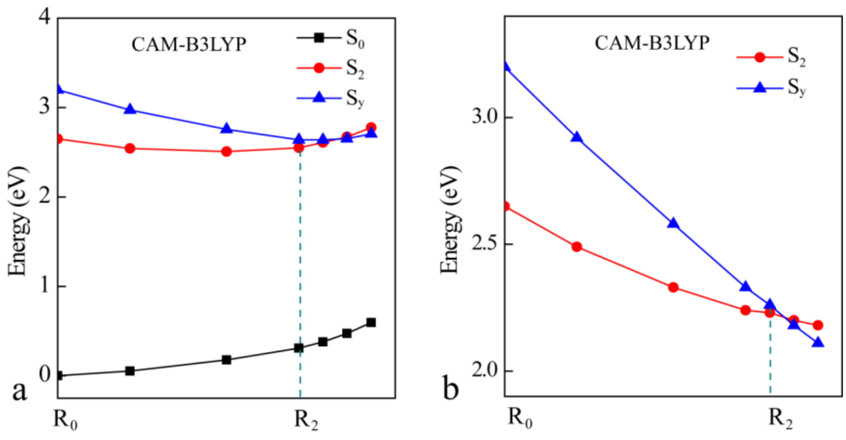

Supplementary Figure 8. Energy profiles for the total energies (a) and the excitation energies (b) in the S_2 and S_y states of RG along the reaction coordinate derived from TDDFT with the CAM-B3LYP functional. R_0 is the ground-state geometry optimized by DFT/CAM-B3LYP. R_2 is the equilibrium geometry in the S_2 state optimized by TDDFT/CAM-B3LYP. Excitation energies in (b) are calculated by BSE. Total energies for the S_2 and S_y states in (a) are the sum of BSE excitation energies in (b) and the DFT ground-state (S_0) energies.

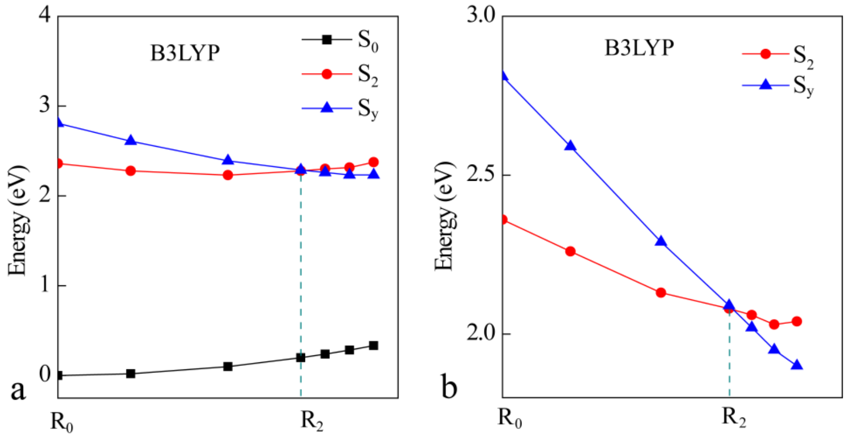

Supplementary Figure 9. Energy profiles for the total energies (a) and the excitation energies (b) in the S_2 and S_y states of RG along the reaction coordinate derived from TDDFT with the B3LYP functional. R_0 is the ground-state geometry optimized by DFT/B3LYP. R_2 is the equilibrium geometry in the S_2 state optimized by TDDFT/B3LYP. Excitation energies in (b) are calculated by BSE. Total energies for the S_2 and S_y states in (a) are the sum of BSE excitation energies in (b) and the DFT ground-state (S_0) energies.

Supplementary Note 4:

Evolvement of S_2 and S_y energies along TDDFT reaction pathways

To further support our conclusions present in the main context that the emission energy of the S_y state is lower than that of the S_2 state and the S_2 state can relax into the S_y state nonadiabatically, we also exam the excited-state dynamics of RG with the aid of TDDFT for excited-state structures optimization. Figure 8 and 9 display the variation of excitation energies and total energies in the S_2 and S_y states with the CAM-B3LYP and B3LYP functionals respectively. In these two runs, R_0 is the ground-state equilibrium geometry optimized by DFT/CAM-B3LYP and DFT/B3LYP, R_2 is the equilibrium geometry in the S_2 state optimized by TDDFT/CAM-B3LYP and TDDFT/B3LYP. TDDFT may have relatively large error in excitation energy. However it might provide reasonable prediction on the structure change in the excited state, especially for the S_2 state of Cars whose composition is simple (i.e. HOMO→LUMO predominately). Due to the low computational cost compared to other high-level quantum chemistry methods, together with the analytic excited-state energy gradient available, TDDFT is a suitable choice to relax Cars in the excited state. However, in Figure 8 and 9 all the excitation energies are recalculated by BSE to give more accurate energies. The total energies for the S_2 and S_y states in Figure 8 and 9 are got by summing these BSE excitation energies and the ground-state energies. This is the reason why the minima of the energy curves for the S_2 state in Figure 8 (a) and 9 (a) do not coincide exactly with the R_2 positions. Nevertheless, these minima do not deviate too much from R_2.

In Figure 8 (9) for the CAM-B3LYP (B3LYP) functional, the S_2-S_y energy gap at R_0 is 0.55 (0.45) eV, the S_2 excitation energy downshifts by 0.41 (0.28) eV from R_0 to R_2, and the S_y excitation energy redshifts by 0.93 (0.72) eV from R_0 to R_2. Although there is a large difference in these physical quantities between the two functionals, the energies, including both the excitation energy and total energy, in the S_2 and S_y states are very close at R_2 for these two functionals. There is an apparent energy surface crossing between the S_2 and S_y states. S_y reaches its own potential minimum soon after passing the R_2 geometry. As a consequence, the emission energy of S_y is a little bit (0.1~0.2 eV) lower than that of S_2. This is consistent with the results obtained from the CDFT-derived excited-state dynamics in the main context. Overall, our conclusions that S_2 can decay into S_y and the emission energy of S_y is lower than S_2 are robust against the difference in the technique to relax RG in the excited state. These support the experimental findings of the new state in two-dimensional electronic spectroscopy (2DES) and high time resolution broadband pump–probe spectroscopy. The stretching modes of C-C and C=C are 1195 and 1590 cm^{-1}, respectively[24]. Although the crossing

point between the S_2 and S_y states is not in the R_0-R_2 region, vibration can assist the transition from S_2 to S_y.

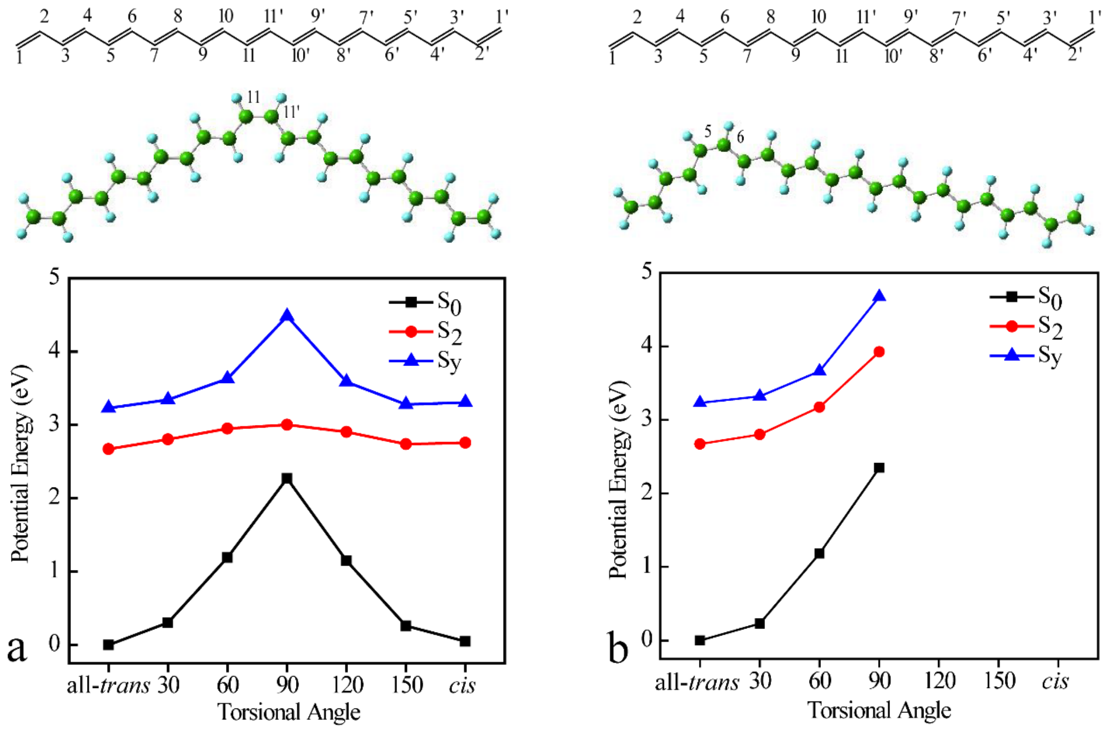

Supplementary Figure 10. Potential surfaces for the S_0, S_2 and S_y states of the N=11 polyene along the torsional coordinate. **(a)** from the all-*trans* (top) to the 11-*cis* (middle) configurations. **(b)** from the all-*trans* (top) to the 5-*cis* (middle) configurations. Since potential surfaces are nearly symmetric with respect to the 90° configuration, as can be seen from (a), only the surfaces from 0°~90° are depicted in (b). In these two calculations, the polyene is twisted around one C=C bond rigidly with the bond lengths and bond angles fixed at their ground-state values. Please note that since bond length alternation is not considered, there is no minimum in the S_2 and S_y potential surfaces in (a) and (b). This is different from the protonated Schiff bases with N=5 whose lowest minimum for the first bright state locates at the torsional angle around 90° as proved in experiments[25] and our previous GW-BSE calculations[26].

Supplementary References

1. Ostroumov, E., Müller, M.G., Marian, C.M., Kleinschmidt, M. & Holzwarth, A.R. Electronic Coherence Provides a Direct Proof for Energy-Level Crossing in Photoexcited Lutein and β-Carotene. *Phys. Rev. Lett.* **103**, 108302 (2009).
2. Tavan, P. & Schulten, K. Electronic excitations in finite and infinite polyenes. *Phys. Rev. B* **36**, 4337-4358 (1987).
3. Cherezov, V., Clogston, J., Papiz, M.Z. & Caffrey, M. Room to Move: Crystallizing Membrane Proteins in Swollen Lipidic Mesophases. *J. Mol. Biol.* **357**, 1605-1618 (2006).

4. Frisch, M.J., Trucks, G. W., Schlegel, H. B., Scuseria, G. E., Robb, M. A., Cheeseman, J. R., Scalmani, G., Barone, V., Mennucci, B., Petersson, G. A., Nakatsuji, H., Caricato, M., Li, X., Hratchian, H. P.,Izmaylov, A. F., Bloino, J., Zheng, G., Sonnenberg, J. L., Hada, M., Ehara, M., Toyota, K., Fukuda, R., Hasegawa, J., Ishida, M., Nakajima, T., Honda, Y., Kitao, O., Nakai, H., Vreven, T., Montgomery, J. A., Peralta, J. E., Ogliaro, F., Bearpark, M., Heyd, J. J., Brothers, E., Kudin, K. N., Staroverov, V. N., Kobayashi, R., Normand, J., Raghavachari, K., Rendell, A., Burant, J. C., Iyengar, S. S., Tomasi, J., Cossi, M., Rega, N., Millam, J. M., Klene, M., Knox, J. E., Cross, J. B., Bakken, V., Adamo, C., Jaramillo, J., Gomperts, R., Stratmann, R. E., Yazyev, O., Austin, A. J., Cammi, R., Pomelli, C., Ochterski, J. W., Martin, R. L., Morokuma, K., Zakrzewski, V. G., Voth, G. A., Salvador, P., Dannenberg, J. J., Dapprich, S., Daniels, A. D., Farkas, Foresman, J. B., Ortiz, J. V., Cioslowski, J., and Fox, D. J *Gaussian 09, Revision B.01*, Gaussian, Inc, Wallingford, CT, (2010).
5. Lindal, T. & Liaaen-Jensen, S. Bacterial carotenoids 56. On the spirilloxanthin stereoisomeric set. *Acta Chem. Scand.* **51**, 1128-1131 (1997).
6. Hengartner, U., Bernhard, K., Meyer, K., Englert, G. & Glinz, E. Synthesis, Isolation, and NMR‐Spectroscopic Characterization of Fourteen (Z)‐Isomers of Lycopene and of Some Acetylenic Didehydro‐and Tetradehydrolycopenes. *Helv. Chim. Acta* **75**, 1848-1865 (1992).
7. Hu, Y., Hashimoto, H., Moine, G., Hengartner, U. & Koyama, Y. Unique properties of the 11-cis and 11, 11′-di-cis isomers of β-carotene as revealed by electronic absorption, resonance Raman and 1 H and 13 C NMR spectroscopy and by HPLC analysis of their thermal isomerization. *J. Chem. Soc., Perkin Trans. 2*, 2699-2710 (1997).
8. Fujii, R., Chen, C.-H., Mizoguchi, T. & Koyama, Y. 1 H NMR, electronic-absorption and resonance-Raman spectra of isomeric okenone as compared with those of isomeric β-carotene, canthaxanthin, β-apo-8′-carotenal and spheroidene. *Spectrochim. Acta, Part A* **54**, 727-743 (1998).
9. Christensen, R.L. et al. Energetics and dynamics of the low-lying electronic states of constrained polyenes: Implications for infinite polyenes. *J. Phys. Chem. A* **117**, 1449-1465 (2013).
10. Shreve, A. et al. On subpicosecond excitation energy transfer in light harvesting complexes (LHC): the B800–850 LHC of Rhodobacter sphaeroides 2.4. 1. *J. Lumin.* **53**, 179-186 (1992).
11. Polívka, T. & Sundström, V. Ultrafast Dynamics of Carotenoid Excited States−From Solution to Natural and Artificial Systems. *Chem. Rev.* **104**, 2021-2072 (2004).
12. Jiang, Y.S. et al. Isolation by high‐pressure liquid chromatography, configurational determination by 1H‐NMR, and analyses of electronic absorption and raman spectra of isomeric spheroidene. *Biospectroscopy* **2**, 47-58 (1996).
13. Coccia, E., Varsano, D. & Guidoni, L. Ab Initio Geometry and Bright Excitation of Carotenoids: Quantum Monte Carlo and Many Body Green's Function Theory Calculations on Peridinin. *J. Chem. Theory Comput.* **10**, 501-506 (2014).
14. Wagner, N.L., Greco, J.A., Enriquez, M.M., Frank, H.A. & Birge, R.R. The nature of the intramolecular charge transfer state in peridinin. *Biophys. J.* **104**, 1314-1325 (2013).
15. Enriquez, M.M. et al. Effect of molecular symmetry on the spectra and dynamics of the intramolecular charge transfer (ICT) state of peridinin. *J. Phys. Chem. B* **116**, 10748-10756 (2012).
16. Enriquez, M.M. et al. The intramolecular charge transfer state in carbonyl-containing polyenes and carotenoids. *J. Phys. Chem. B* **114**, 12416-12426 (2010).
17. Stanton, J.F., Gauss, J., Ishikawa, N. & Head‐Gordon, M. A comparison of single reference methods for characterizing stationary points of excited state potential energy surfaces. *J. Chem. Phys.* **103**, 4160-4174 (1995).
18. McCarthy, M., Rosmus, P., Werner, H.J., Botschwina, P. & Vaida, V. Dissociation of NH3 to NH2+ H. *J. Chem. Phys.* **86**, 6693-6700 (1987).

19. Linstrom, P.J. & Mallard, W. NIST Chemistry webbook; NIST standard reference database No. 69. (2001).
20. Herzberg, G. Electronic spectra and electronic structure of polyatomic molecules, Vol. 2. (Krieger Publishing Company, 1991).
21. Rohlfing, M. & Louie, S.G. Electron-hole excitations and optical spectra from first principles. *Phys. Rev. B* **62**, 4927-4944 (2000).
22. Onida, G., Reining, L. & Rubio, A. Electronic excitations: density-functional versus many-body Green's-function approaches. *Rev. Mod. Phys.* **74**, 601-659 (2002).
23. Ismail-Beigi, S. & Louie, S.G. Excited-State Forces within a First-Principles Green's Function Formalism. *Phys. Rev. Lett.* **90**, 076401 (2003).
24. Ostroumov, E.E., Mulvaney, R.M., Cogdell, R.J. & Scholes, G.D. Broadband 2D Electronic Spectroscopy Reveals a Carotenoid Dark State in Purple Bacteria. *Science* **340**, 52-56 (2013).
25. Liebel, M. et al. Direct observation of the coherent nuclear response after the absorption of a photon. *Phys. Rev. Lett.* **112**, 238301 (2014).
26. Kaczmarski, M.S., Ma, Y. & Rohlfing, M. Diabatic states of a photoexcited retinal chromophore from ab initio many-body perturbation theory. *Phys. Rev. B* **81**, 115433 (2010).
